# Effectiveness of complete conservative treatment for adolescent idiopathic scoliosis (bracing and exercises) based on SOSORT management criteria: results according to the SRS criteria for bracing studies - SOSORT Award 2009 Winner

**DOI:** 10.1186/1748-7161-4-19

**Published:** 2009-09-04

**Authors:** Stefano Negrini, Salvatore Atanasio, Claudia Fusco, Fabio Zaina

**Affiliations:** 1 ISICO (Italian Scientific Spine Institute), Via Roberto Bellarmino 13/1, 20141 Milan, Italy

## Abstract

**Background:**

The SRS criteria give the methodological reference framework for the presentation of bracing results, while the SOSORT criteria give the clinical reference framework for an appropriate bracing treatment. The two have not been combined in a study until now. Our aim was to verify the efficacy of a complete, conservative treatment of Adolescent Idiopathic Scoliosis (AIS)according to the best methodological and management criteria defined in the literature.

**Methods:**

Study Design. Retrospective study. Population. We included all AIS patients respecting the SRS inclusion criteria (age 10 years or older; Risser test 0-2; Cobb degrees 25-40°; no prior treatment; less than one year post-menarchal) who had reached the end of treatment since our institute database start in 2003. Thus we had 44 females and four males, with an age of 12.8 ± 1.6 at the commencement of the study. Methods. According to individual needs, two patients have been treated with Risser casts followed by Lyon brace, 40 with Lyon or SPoRT braces (14 for 23 hours per day, 23 for 21 h/d, and seven for 18 h/d at start), and two with exercises only (1 male, 1 female): these were excluded from further analysis. Outcome criteria. SRS (unchanged; worsened 6° or more; over 45° at the end of treatment; surgically treated; two years' follow-up); clinical (ATR, Aesthetic Index, plumbline distances); radiographic (Cobb degrees); and ISICO (optimal; minimal). Statistics. Paired ANOVA and t-test, Tukey-Kramer and chi-square test.

**Results:**

Median reported compliance during the 4.2 ± 1.4 treatment years was 90% (range 5-106%). No patient progressed beyond 45°, nor was any patient fused, and this remained true at the two-year follow-up for the 85% that reached it. Only two patients (4%) worsened, both with single thoracic curve, 25-30° Cobb and Risser 0 at the start. We found statistically significant reductions of the scoliosis curvatures (-7.1°): thoracic (-7.3°), thoracolumbar (-8.4°) and lumbar (-7.8°), but not double major. Statistically significant improvements have also been found for aesthetics and ATR.

**Conclusion:**

Respecting also SOSORT management criteria and thus increasing compliance, the results of conservative treatment were much better than what had previously been reported in the literature using SRS criteria only.

## Background

Little evidence currently exists in regard to bracing for AIS (Adolescent Idiopathic Scoliosis). There are only two solid studies published in the literature: a controlled observational trial by Nachemson et al[1] gives results in favour of bracing; the Wong et al. [2] randomised controlled study suggests the superiority of a rigid TLSO over the SpineCor brace. Apart from these studies, there are numerous case series having certain historical controls: Recently these have been partially summarised in a systematic review [3], from which the papers including exercises and those not published in English literature had been excluded [4-7]. Considering the surgery rates in curvatures between 20° and 45° at the start of treatment, the results varied greatly: in a total of 1814 patients, two papers had rates below 10%, six between 11% and 20%, two between 21% and 30%, five between 31% and 40%, and one exceeding 41%. Given such an extent of variation, there was no difference found by comparison with the natural history papers (139 patients), in which two out of three reported a 13% surgery rate, and the other 38.3% [3]. This big variability of results of bracing can have many causes, including methodological bias, quality of bracing and compliance.

Today we have some instruments published in the literature to face these problems, which could lead to better papers than before. The SRS criteria [8] give the methodological reference framework by which to select the study population and produce results so as to make them comparable among studies, and in this way some methodological biases should be overcome. The SOSORT criteria [9] give the clinical reference framework for an appropriate bracing treatment. In this way compliance should be increased, and the quality of bracing should be improved as well. Today, papers which consider these criteria should offer the best combination of methodological and clinical quality.

Until now, no paper has been published with respect to the SOSORT criteria, but two papers have followed the SRS criteria. According to a retrospective study by Janicki et al. [10], a TLSO leads to a 79% rate of fusion while the Providence brace leads to 60%; in a prospective study, Coillard [11] reported a 22.9% rate of fusion with the SpineCor brace, which dropped to 18.1% with an increased population, according to the last abstract presented during the 2009 SOSORT Meeting [12]. If respecting only methodological criteria, the great variability of results seems to remain an issue.

In 2008 we published a retrospective study on a prospective database [7] reporting our own results in a population of 112 AIS patients, 13.2 ± 1.8 years old, with 23.4 ± 11.5° Cobb degrees at the start of treatment. The rates of surgery were 0.9% (efficacy analysis), and 4.5% (worst case). Overall, the curvatures exceeding 40°, which numbered 11 at the start of observation, were reduced to three at the end. This study did not consider the SRS criteria, so therefore we must again verify our results according to these standards, considering that our clinical practice has for many years been carried out with full consideration of the SOSORT criteria [9].

The aim of this paper was to verify the efficacy of a complete, conservative treatment of AIS by following the SRS and SOSORT criteria.

## Methods

### Study design

This is a retrospective study on a prospective database that started in March 2003, including all visits performed since September 2003 at our institute. When the study was carried out, 6,172 patients and 21,024 evaluations had been included in the database, 3,937 patients had idiopathic scoliosis, and 685 had reached the end of treatment.

### Population

According to the SRS paper on brace studies [8], inclusion criteria at the start of treatment were: AIS; age 10 years or older; Risser test 0-2; Cobb degrees 25-40°; no prior treatment; and less than one year post-menarchal. All patients who satisfied the inclusion criteria at the start and had finished their treatment were considered in the study.

We had 44 females and four males; at the start the age was 12.8 ± 1.6 years, while Cobb degrees were 30.4 ± 4.4°. According to SRS criteria, we had subgroups for curvature types, curve magnitude and skeletal maturity (Table 1). We performed subgroup analysis for gender.

**Table 1:** Baseline characteristics of the entire sample and the identified sub-groups.

Sub-groups		Abbreviation	Number	F/M	Age	BMI	°Cobb	AI	ATR
	**Total braced**		46	43/1	12.8 ± 1.5	18.7 ± 2.6	30.4 ± 4.4	3.4 (0-6)	7.8 ± 4.1

	**Exercises**		2	1/1	11.6 ± 1.9	26.7*	26.0 ± 1.1	3*	4.5*

**Curvature type**	**Thoracic**	TH	13	12/1	13.2 ± 1.9	19.2 ± 1.4	31.8 ± 4.3	4.2 (2-6)	8.3 ± 4.1

	**Thoracolumbar**	TL	5	5/0	12.3 ± 1.3	18.3 ± 1.8	33.4 ± 4.8	3.0 (2-4)	11.2 ± 2.5

	**Lumbar**	LU	16	15/1	13.0 ± 1.5	18.2 ± 2.4	29.6 ± 4.3	2.5 (0-4)	5.9 ± 3.5

	**Double major**	DM	13	11/1	12.5 ± 1.4	19.6 ± 5	28.7 ± 3.9	4.0 (2-6)	8.4 ± 4.9

	**P**			NS	NS	NS	TL>DM	T>TL DM>L	TL>L

**Magnitude of curvature**	**25-30**	S	24	23/1	12.5 ± 1.4	18.7 ± 3.1	27.0 ± 1.9	3.1 ± 1.5	6.8 ± 4.5

	**31-35**	M	15	13/2	13.0 ± 1.6	18.8 ± 1.3	33.0 ± 2.5	3.8 ± 1.5	8.9 ± 3.7

	**36-40**	L	7	7/0	13.7 ± 2.0	18.5 ± 2.4	37.7 ± 1.6	3.5 ± 1.4	8.7 ± 3.4

	**P**			NS	NS	NS	-	NS	NS

**Skeletal maturity**	**Risser 0**	R0	28	27/2	12.4 ± 1.5	17.9 ± 2.3	29.4 ± 4.2	3.5 ± 1.5	7.7 ± 4.1

	**Risser 1**	R1	6	6/1	12.4 ± 1.2	20.6 ± 3.7	33.3 ± 4.0	3.8 ± 0.8	10.1 ± 4.1

	**Risser 2**	R2	12	11/1	14.1 ± 1.5	18.8 ± 1.3	31.3 ± 4.5	2.9 ± 1.8	6.7 ± 4.1

	**P**			NS	R2>R0 R2>R1	NS	R1>R0	NS	NS

**Treatment**	**Risser cast**	RC	2	2/0	11.4 ± 0.1	-	29.5 ± 6.4	6*	5*

	**Brace 23 hours/day**	B23	14	13/1	13.2 ± 1.7	18.3 ± 2.4	34.5 ± 3.9	3.8 ± 1.4	9.8 ± 4.4

	**Brace 21 hours/day**	B21	23	22/1	13.0 ± 1.5	18.5 ± 1.9	28.2 ± 3.3	2.9 ± 1.5	6.5 ± 3.9

	**Brace 18 hours/day**	B18	7	6/1	12.4 ± 1.8	17.8 ± 0.9	31.1 ± 2.7	3.3 ± 1.1	9.0 ± 0.9

	**P**			NS	NS	NS	C23>C21 C18>C21	NS	C23>C21

NS: not significant. * only one value.

### Treatments

**Figure 1:**
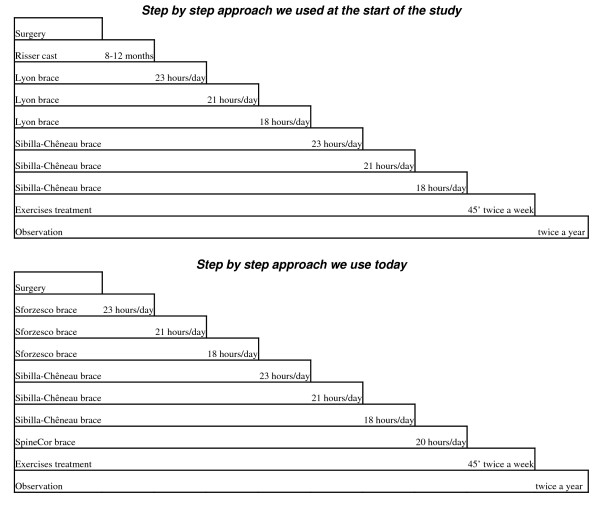
**Step-by-step theory**. The step-by-step Sibilla theory [13].

In our everyday clinic, according to a complete evidence-based clinical practice, joining evidence to clinical expertise and patients' preferences, we do not set standard treatment methods [7,13]. Consequently, the patients included in this study were treated on an individual basis according to their needs, and a therapeutic contract was established at each visit with the patient and his/her family. We apply a full set of conservative treatments, including exercises and braces: elastic (SpineCor since two years), rigid (Sibilla-Cheneau brace) and very rigid (Sforzesco brace, but also Risser cast until three years ago). We follow the "step-by-step" Sibilla theory of treatment of scoliosis [13-15]. in which each step represents an increase not only in the strength of treatment but also in regard to the requirements placed on the patient (Figure 1).

Most of the patients respecting the SRS inclusion criteria (44) had been treated with braces full-time (from 18 to 23 hours per day) until they reached Risser 3 when, according to our protocol, brace weaning started and lasted an average 2.2 years until Risser 5 [16]. According to the individual needs, and respecting a discussion with the patient and family so as to maximally increase compliance, we defined the hours per day of bracing in each single child: 23 (14 patients), 21 (23 patients) or 18 (seven patients) hours per day. In this retrospective case series based on the SRS criteria, we also had two patients that had been treated with eight months of Risser cast, followed by Lyon brace, and two treated with exercises only: these have been excluded from further analysis and considered as a separated group. The characteristics of the patients according to the treatment proposed are listed in Table 1.

During treatment we follow the SOSORT criteria for several years. In the Additional File 1 the answer to the questionnaire for clinical studies proposed by SOSORT is reported [9]. According to the classification proposed, we had an excellent approach, with 43 of 44 questions receiving "yes" responses.

The treatments used in this study have been carefully described in an online open-access booklet http://www.isico.it/approach/default.htm[13]. A short description follows:

#### Brace treatment

The braces we used, obviously always adapted according to the curve patterns, were chosen on the basis of individual needs. The following were included [7,13]: Risser cast and Lyon, or Sforzesco-SPoRT brace for the most important cases; in cases where reduced forces were required, we used the Sibilla-Chêneau brace for thoracic, thoracolumbar and double-major curvatures, and the Lapadula for thoraco-lumbar and lumbar ones. During these years we gradually changed our mechanical approach to bracing from a mainly three-point system to the SPoRT concept [17,18], and therefore patients have been treated according to both approaches.

The goal of brace treatment varied according to the degree of curvature considered, and the forces (in terms of the type of brace and hours of usage) were consequently administered (Figure 1). The weaning period [13,16] required a gradual increase in the hours without the brace while allowing the patient to maintain the correction thus achieved. This is why we reduced the wearing of the brace by no more than two or three hours every six months, and why the stabilization exercises were considered so crucial during this period.

#### Exercise treatment

Exercises varied according to the stage of treatment [13].

In two patients they were performed as the only treatment to avoid bracing [19]: in these cases active self-correction [20] was the key movement required while performing stabilization, strengthening while exercises aimed at increasing the range of motion have been avoided.

In all the other patients, who wore braces, the aims of exercises were to increase the correction allowed by the brace, and avoid the loss of correction while weaning the brace [13]. To enhance the correction, a set of mobilization exercises was proposed for two months in preparation for bracing (before wearing the brace and in the first weeks of bracing) [21]; afterwards, exercises to increase the corrective forces of bracing [22], together with mobilization (increase of range of motion) and strengthening, were proposed. When starting the weaning phase, exercises gradually changed to those performed by patients not wearing the brace [16].

### Outcome criteria

According to the SRS criteria [8], we verified the percentage of patients unchanged, worsened 6° or more, exceeding 45° at the end of treatment, and fused, with the required two years of follow-up in 40 patients (85%). For this study we also had radiographic (Cobb degrees) and clinical results (ATR, Aesthetic Index, plumbline distances), which were also considered in terms of the percentage of change among patients over the repeatability error.

Additionally, we propose the ISICO outcomes [7], which we use in our everyday clinic (Table 2); in fact, we usually define individual outcomes case by case, according to a general medical reference setting and a criterion for the acceptability of the patient and his/her family. The ISICO outcomes can be divided into absolute (avoiding surgery), minimal and optimal. The latter are based on data from the literature which indicates the need to be as far as we can from the two recognized thresholds of scoliosis (50 degrees, i.e. the near certainty of progression in adulthood; and 30 degrees, i.e. the possibility of progression) [23]. Given these goals, in everyday clinics we continuously adapt ourselves according to what we obtain, as well as to how the patient behaves and feels. We establish and constantly renew the contract with the patient and his/her parents, who in this way are fully integrated within the rehabilitation team. This allows one to obtain some minimal results even in the most difficult patients, particularly with those who do not comply with our prescriptions for best results.

**Table 2:** The ISICO outcomes [7]

	Minimal	Optimal	Absolute
**25-29°**	<30°	<25°	Avoiding surgery
	
**30-40°**	stable	<30°	

The ISICO outcomes are established during treatment for each single patient according to the starting curvature. The absolute aim is for all patients to avoid surgery, but we also have the goal of obtaining an optimal result as stated in the table. When difficulties arise, and compliance decreases, or the curvature offers higher resistance to treatment, a minimal outcome is in any case anticipated. Thus the table indicate only the criteria regarding the ranges of curvature considered in this paper.

Reported compliance has also been considered. Each patient and his or her parents was carefully queried at each visit about how many hours per day the patient had used the brace, as well as the average usage during the period reported. This was compared with the prescription, and a percentage of compliance was computed.

### Computations and statistical analyses

We used the paired ANOVA, the Tukey-Kramer test, the paired t-test and chi-square analysis according to what was appropriate. Evaluating the percentage of patients changed, we considered significant clinical changes if the repeatability error was exceeded, namely:

• Cobb degrees: 5° [24]

• ATR: 2° [25]

• Aesthetic Index (AI): 2 points [26]

• Plumbline distances: 10 mm for C7, 15 for L3, and 20 for Sagittal Index (SI = C7+L3) [24]

## Results

At baseline the subgroups of patients were statistically different for some characteristics (Table 1).

Treatment lasted 4.2 ± 1.4 years with no differences among the subgroups. The median reported compliance has been 90% (range 5-106%); 30% of patients reported compliance of 100% or more, and 90% reported at least 80%; we found no significant difference among the sub-groups for this parameter.

### SRS Criteria Outcomes

No patient progressed beyond 45°, nor was anyone fused, and this remained true in the 85% of patients who reached the two years' follow-up (Table 3). Due to our results, we decided to add the outcome "improved" for patients whose Cobb degrees were reduced 6° or more. Generally, the number of patients who had improved was higher than those who experienced no change, with the few exceptions of double major, 36-40° and Risser 1 subgroups. We had only two patients who worsened, and this should serve to interpret the results in the subgroups cautiously; in fact, both patients had thoracic curvatures with a magnitude between 25° and 30°, and were Risser 0, while they were treated with the brace for 23 or 21 hours per day.

**Table 3:** Results according to the SRS outcome criteria.

Sub-groups		Unchanged (improved)	Worsened	Over 45° EOT	Surgery EOT	Surgery FU
	**Total braced**	96% (59%)	4%	0	0	0

	**Exercises**	100% (0)	0	0	0	0

**Curvature type**	**Thoracic**	86% (50%)	14%	0	0	0

	**Thoracolumbar**	100% (80%)	0	0	0	0

	**Lumbar**	100% (88%)	0	0	0	0

	**Double major**	100% (15%)	0	0	0	0

**Magnitude of curvature**	**25-30**	92% (54%)	8%	0	0	0

	**31-35**	100% (67%)	0	0	0	0

	**36-40**	100% (43%)	0	0	0	0

**Skeletal maturity**	**Risser 0**	93% (59%)	7%	0	0	0

	**Risser 1**	100% (29%)	0	0	0	0

	**Risser 2**	100% (67%)	0	0	0	0

**Treatment**	**Risser cast**	100% (50%)	0	0	0	0

	**Brace 23 hours/day**	93% (50%)	7%	0	0	0

	**Brace 21 hours/day**	96% (65%)	4%	0	0	0

	**Brace 18 hours/day**	100% (57%)	0	0	0	0

EOT: End of treatment; FU: 2 years' follow-up.

Due to our results, we added (in brackets) the percentages of "improved" patients, including those in which Cobb degrees were reduced 6° Cobb or more. Due to the fact that we had only two patients worsened, the results in the subgroups should be considered cautiously. In fact, both patients had thoracic curvatures of magnitude between 25° and 30° and were Risser 0, while they were treated with the brace for 23 or 21 hours daily.

### Clinical and radiographic outcomes

**Figure 2:**
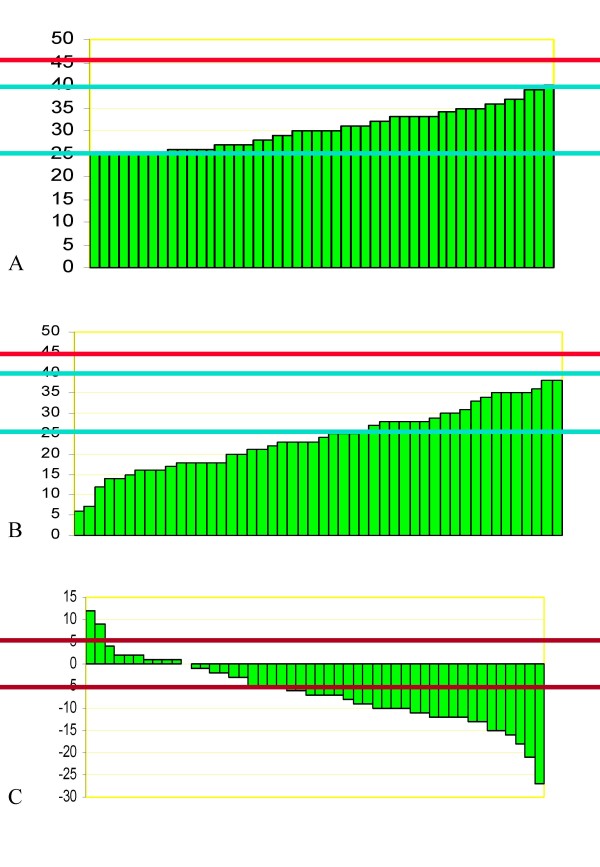
**Individual patients radiographic results**. A. Starting data (Cobb degrees) of the patients included in this study. B. Final radiographic results (Cobb degrees) of each single patient included in the study. C. Results of brace treatment in all patients included in the study: 2 patients worsened, 18 improved. The blue lines represent the SRS inclusion criteria (from 25 to 40 Cobb degrees), while the red ones report the final SRS end-point not to be reached at the end of treatment (45 Cobb degrees). The maroon lines indicate the ± 5 Cobb degrees that represents the significant limit to achieve a clinical change in single patients.

**Figure 3:**
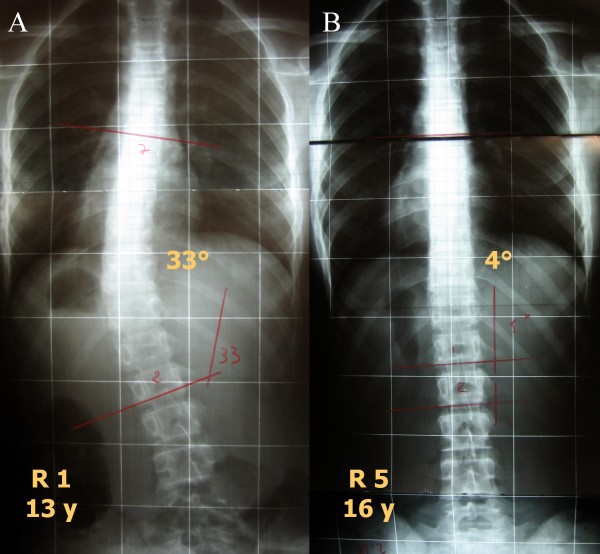
**One of the best results obtained in this study**. A. Starting x rays. B. Final x-rays. P decreased from 33° at 13 years of age, Risser 1, pre-menarchal to 4° at 16 years, Risser 5.

**Figure 4:**
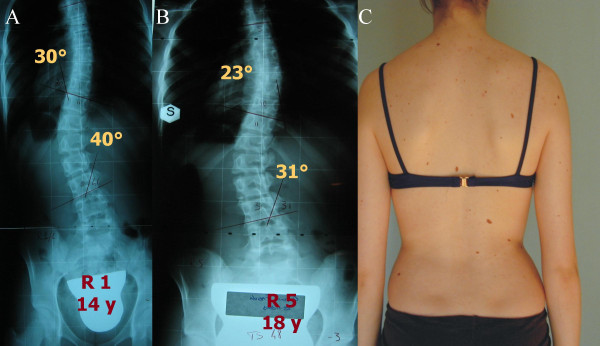
**Typical result in this study**. A. Starting x rays. B. Final x-rays. C. Aesthetic final result. F decreased from 30-40° at 14 years of age, Risser 1, pre-menarchal to 23-31° at 18 years, Risser 5.

**Figure 5:**
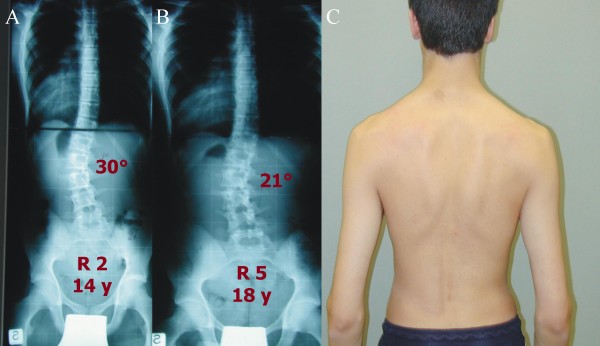
**Results of a male patient**. A. Starting x rays. B. Final x-rays. C. Aesthetic final result. C decreased from 30° at 14 years of age, Risser 2 to 21° at 18 years, Risser 5.

We found highly statistically significant reductions of Cobb degrees (Figure 2), aesthetics and ATR (Table 4), but we also had a statistically significant negative impact on the sagittal profile for C7 (-7.7 mm) and SI (-13.0 mm). Clinically, no patient worsened for ATR; for all the other parameters, fewer than 10% of patients worsened; on the contrary, improvements were very common, at up to 100% (thoraco-lumbar curvatures' ATR). Sub-grouping only showed that double-major and thoracic curvatures had worse results; the Risser cast and exercise sub-groups were too small to allow statistical analysis. In Figure 3, 4 and 5 single clinical cases are reported.

**Table 4:** Clinical and radiographic results.

		Cobb degrees	Angle of Trunk Rotation (ATR)				Aesthetic Index (AI)			
**Sub-groups**		**Av**	**Av**	**I**	**U**	**W**	**Av**	**I**	**U**	**W**

	**Total braced**	-7.3 (8.4) *	-3.4 (3.9) *	35%	65%	0	-1.6 (1.8) *	45%	52%	3%

	**Exercises**	+1.5 (0.7)	2.0^§^	0	100%	0	-1^§^	0	100%	0

**Curvature type**	**Thoracic**	-6.3 (13.0)	-2.4 (4.4)	43%	57%	0	-2.2 (1.9) *	55%	45%	0

	**Thoracolumbar**	-8.8 (7.0) *	-8.1 (2.0) *	100%	0	0	-1.0 (2.0)	25%	75%	0

	**Lumbar**	-10.4 (3.7) *	-2.5 (3.6) *	18%	82%	0	-1.0 (1.7)	42%	50%	8%

	**Double major**	-2.8 (5.3)	-3.1 (2.5) *	33%	67%	0	-1.7 (1.6)	50%	50%	0

**Magnitude of curvature**	**25-30**	-5.6 (7.6) *	-2.5 (3.9) *	31%	69%	0	-1.2 (1.9) *	41%	53%	6%

	**31-35**	-9.7 (10.1) *	-4.1 (4.3) *	40%	60%	0	-2.3 (1.6) *	60%	40%	0

	**36-40**	-6.3 (7.4) *	-4.1 (3.4) *	40%	60%	0	-1.1 (1.4)	33%	67%	0

**Skeletal maturity**	**Risser 0**	-6.6 (9.6) *	-3.3 (3.9) *	26%	74%	0	-1.6 (2.1) *	45%	50%	5%

	**Risser 1**	-4.0 (5.7)	-3.6 (3.5)	33%	67%	0	-1.5 (1.8)	40%	60%	0

	**Risser 2**	-9.7 (6.0) *	-2.9 (4.6)	50%	50%	0	-1.4 (1.0) *	50%	50%	0

**Treatment**	**Risser cast**	-15.0 (26.9)	-2.5^§^	0	100%	0	-5^§^	100%	0	0

	**Brace 23 hours/day**	-6.4 (9.8) *	-4.2 (3.5) *	33%	67%	0	-1.5 (1.7) *	33%	67%	0

	**Brace 21 hours/day**	-7.5 (6.4) *	-2.7 (4.5) *	38%	62%	0	-1.2 (1.8) *	50%	44	6%

	**Brace 18 hours/day**	-6.6 (5.4) *	-4.8 (2.0)	50%	50%	0	-2.2 (1.9)	67%	33%	0

	**Exercises**	+1.5 (0.7)	2.0^§^	0	100%	0	-1^§^	0	100%	0

Av: Average (Standard Deviation); I: Improved; U: Unchanged; W: Worsened; ATR: Angle of Trunk Rotation according to Bunnel; AI: Aesthetic Index. *: Statistically significant difference, paired t-test pre-post treatment; §: Only one patient.

Average improvements were statistically significant in nearly all parameters. Generally speaking, the double-major and thoracic curvatures had the worst results, while the Risser cast and exercise sub-groups were too small to allow any statistical analysis. These treatments are generally used in curvatures which are respectively greater and lower than those considered in this population, where they have been present because of the evidence-based clinical practice approach used in this study.

### ISICO Outcomes

**Figure 6:**
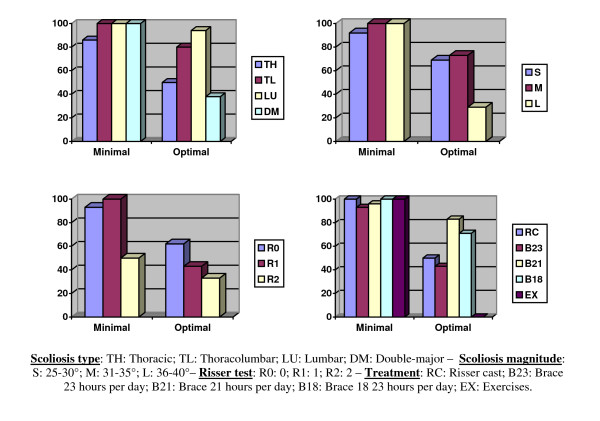
**ISICO outcomes**. The ISICO outcomes [7] are a representation of the evidence-based clinical practice approach used in this paper. According to individual needs, based on the starting x-rays, a radiographic desired optimal result is defined (Table 2); according to this starting criterion and to what is gradually obtained, based on compliance and curvature resistance to treatment, a minimal criterion can be considered. In these graphs the sub-group analysis are reported. It is easier to obtain optimal results in thoraco-lumbar and lumbar curvatures, in scoliosis up to 35 degrees, and in the youngest patients.

According to the ISICO outcomes, 96% of patients had minimal and 65% optimal results (Figure 6); optimal results were obtained mainly in thoraco-lumbar and lumbar curvatures, in scoliosis up to 35 degrees, and the youngest patients.

In all analysis performed, gender or patients braced only did not show any difference with the total population. The results at 2 years follow-up were not different from those at the end of treatment.

## Discussion

According to this study it is possible, in patients selected according to the SRS inclusion criteria, and treated with an appropriate conservative treatment following SOSORT criteria, to obtain reductions of AIS in most of the patients. This is true considering the SRS outcomes but also in regard others such as the Cobb degrees, ATR, Aesthetic Index and ISICO clinical outcomes. Moreover, in patients who accept treatment it is possible to avoid surgery in AIS that has not previously been treated, with curvatures ranging from 25° to 40° and Risser between 0 and 2.

The subject study was retrospective, and therefore it includes only an efficacy analysis of patients who had reached the end of treatment. Accordingly, the results should be interpreted from this perspective. Most of the studies published in the literature are retrospective as well [3]; one of the published papers that followed the SRS criteria was retrospective [10], while the other one is prospective [11] and ongoing [12]. Prospective studies allow to perform and intent-to-treat analysis, as suggested by the SRS criteria [8]. Nevertheless, an efficacy analysis has its own value in showing what results can be achieved with patients who follow the required treatment, while, conversely, an intent-to-treat analysis allows one to include all drop-outs, which in any case represent a failure of treatment. What should be questioned is whether drop-outs should be considered as fusions (i.e. real failures) or if, in the case of a conservative approach to AIS, dropping out does not automatically mean the patient will arrive at fusion and/or progress beyond 5°. In fact, in the prospective paper by Coillard, patients who withdraw were listed separately but not included in the fused group [11]. In a previous study [7] we had a low drop-out rate (3.6%, or four patients out of 112) with the same approach presented here, even if the population was different. Currently, we are conducting a prospective study which will presumably be completed within a couple of years' time, in order to perform an intent-to-treat analysis and complete the efficacy analysis performed here.

In this study we did not have one patient fused, nor did we have adolescent who finished treatment at more than 45°. We understand that this result could appear to reduce credibility of the study, being results normally reported in the literature completely different form the ours. Nevertheless, this corresponds to our own everyday experience. Obviously, there could be drop-outs who finish fused, and this can be observed only through a prospective study. Moreover, these results confirm our previous prospective paper published in 2008 [7], where we obtained a surgery rate of 0.9% in this same efficacy analysis (4.5% in the intent to treat, considering drop-outs as fused patients) in a less-specific population including curvatures from 11° to 53° Cobb.

### Comparison with other studies following the SRS criteria

The other two published papers that followed the SRS criteria reported results which were completely different. According to Janicki [10], using a TLSO in 48 AIS, 85% of patients worsened 6° or more, 62.5% were in excess of 45° at the end of treatment, and 79% were fused; using a Providence brace in 35 patients, the outcomes were 69%, 42.8% and 60%, respectively. This was a retrospective paper, and suffered the same possible bias of the study presented here, albeit with a significant difference: Janicki and colleagues are surgeons, while we are conservative specialists. It could be hypothesized that the drop-outs in our series were mainly patient candidates for surgery who did not achieve good results (and this could explain to some extent why we did not have any patient treated surgically in our series) [7], while the opposite could be true for a surgical group (even if we don't believe in this assumption). Moreover, we had a very high compliance rate using braces full-time, while Janicki et al. had the best adherence with nightly bracing. Consequently, they concluded by stating their preference for the latter solution to increase compliance. Nevertheless, we must consider that adherence to treatment is not only a matter of the braces used but also of the total management of patients [9]. Obviously, raising the point of surgeons versus conservative specialists, we are not stating anything about competence, but only about general settings and attitudes toward treatment and interpretation of obtained results, that are possibly understood by patients and can drive their behaviours.

Another significant reason for the differences found with Janicki et al. [10] could be the type of braces used (i.e. mechanisms of action) and/or their quality (i.e. single-brace efficacy in the context of single patients). Unfortunately, we do not have any measurement system for this, apart from in-brace correction [27], which is not reported in these papers.

The other study following SRS criteria, by Coillard et al. [11], was performed on 170 patients wearing the SpineCor brace and reported at the end of treatment (efficacy analysis) that 22.9% of patients were fused, 33.5% had progression of at least 6° and 24.1% finished treatment in excess of 45°. There were 12 patients (7.1%) who withdrew from treatment and were not included in the efficacy analysis. These results were much better than those reported by Janicki [10] but worse than those reported in this study. Nevertheless, the comparison of Coillard's results with ours is coherent with what has been reported elsewhere in the literature. In fact, the effectiveness of the SpineCor brace has been reported to be inferior to rigid TLSO braces in a randomised controlled trial [2], as well as in a study with an historical control group [28].

### Compliance and SOSORT Criteria

The compliance rate can serve as a general justification for these results. SpineCor brace results have been reported by the developers of the brace; moreover, the SpineCor approach requires systematic, frequent contact with the patient by a well-trained team [29]. All these points are part of the SOSORT criteria of brace treatment management [9], which we followed in this study. Consequently, the study by Coillard et al. is more comparable to our results than to those of Janicki, given the management applied during treatment.

Another characteristic of our study should be pointed out: Apart from the application of the SOSORT criteria, each treatment has been tailored on each single patient so as to maximise compliance, as well as to allow the best possible inclusion of patients and their parents in the treating team. Not only the starting point of treatment (23 versus 21, or 18 hours per day of bracing, or even Risser cast or exercises), but also the final possible results were tailored during treatment (i.e. we decreased brace wearing individually, according to the need) in terms of the optimal or minimal results following the ISICO outcomes [7]. Finally, exercises were used as a means to increase compliance, not simply as a way to increase bracing results, as has been proved in some studies [16,21,22]. In practice, all treatment management was focused on the patient not only in terms of SOSORT criteria but also in terms of treatment planning.

Another possible explanation for the high compliance rate observed could be the private setting of our Institute, versus the usual Health National Service one used in the remaining of our country (Italy): nevertheless, in our view the SOSORT criteria, the psychological approach we used, the presence of a complete and well trained team play the most important role in increasing compliance.

### Strengths and weaknesses of the study

This is the third study published with respect to SRS criteria, and it is the first one that has also fulfilled the SOSORT criteria for bracing studies. The former criteria provide the methodological framework while the latter give the clinical framework so as to gather the best possible data on this kind of treatment. The number of patients is low, but the population is comprehensively selected and cohesive.

This is a retrospective study. Ideally, we should have performed a prospective study, but our institute was established in 2003 and we have been collecting a prospective database since that time. We verified, in a preliminary analysis, that the population with respect to the SRS criteria was too low at this stage to perform an adequate prospective study. Consequently, we decided upon a retrospective analysis of all patients who completed their treatment which, at the start, respected the SRS criteria.

Another characteristic of the paper is that not simply has one standardized treatment been proposed but patients have been treated with different braces and some have been treated with exercises exclusively (even if these have been excluded from the whole group and considered as a group per se). Nevertheless, this research concentrates on a complete, conservative approach, focused on the increase of compliance through management and a clinical everyday approach. It represents the everyday clinical reality instead of a "laboratory" setting as it could be in other studies. This could be considered a weakness of the paper as well as a strength.

Other possible limits to be considered include: the high prevalence of females in this population, but this is typical of the everyday clinical reality in scoliosis treatment (moreover we did not find differences according to genders); the inclusion of two patients treated with exercises only and two with casting before bracing, and not only of braced patients, but we did not find any difference in the subgroup analyses, and the retrospective design required in our view to include all patients respecting the SRS criteria without introducing any other possibly confounding inclusion criterion; the fact that only 85% of patients reached the 2 years follow-up, but this subgroup was not different from the entire population for any basal characteristic nor any final result.

It must also be stated that for many years we have conducted our work with consideration for the SOSORT criteria [9], in fact well before they had been established, because they are part of our everyday clinical approach. In this respect, they are not something new to our work, which is totally focused on compliance that exceeds the SOSORT criteria in various respects.

## Conclusion

According to our results, in patients at risk it is possible to avoid surgery, provided the patients follow their prescriptions and adhere to the regimen of treatment. By respecting the SOSORT criteria and focusing on compliance, a complete, conservative treatment based on bracing and exercises will produce results, according to the SRS criteria, which are much better than what has been reported previously. These results should be verified in the future with a prospective paper which also includes drop-outs, which are failures of treatment. This paper demonstrates the importance of the human approach together with the technical aspects of treatment.

## Competing interests

The authors declare that they have no competing interests.

## Authors' contributions

SN, SA, CF and FZ contributed equally to this study. SN treated all patients.

## Supplementary Material

Additional file 1 **Answers to SOSORT Criteria questionnaire**. Answers to the questionnaire to verify the achievement of the SOSORT Criteria for bracing: "Standards of management of idiopathic scoliosis with corrective braces in everyday clinics and in clinical research". Click here for file

## References

[B1] Nachemson AL Peterson LE Effectiveness of treatment with a brace in girls who have adolescent idiopathic scoliosis. A prospective, controlled study based on data from the Brace Study of the Scoliosis ResearchSociety J Bone Joint Surg Am 1995 77 6 815 822 7782353 10.2106/00004623-199506000-00001

[B2] Wong MS Cheng JC Lam TP Ng BK Sin SW Lee-Shum SL Chow DH Tam SY The effect of rigid versus flexible spinal orthosis on the clinical efficacy and acceptance of the patients with adolescent idiopathic scoliosis Spine 2008 33 12 1360 1365 10.1097/BRS.0b013e31817329d9 18496349

[B3] Dolan LA Weinstein SL Surgical rates after observation and bracing for adolescent idiopathic scoliosis: an evidence-based review Spine 2007 32 19 Suppl S91 S100 10.1097/BRS.0b013e318134ead9 17728687

[B4] Rigo M Reiter C Weiss HR Effect of conservative management on the prevalence of surgery in patients with adolescent idiopathicscoliosis Pediatr Rehabil 2003 6 3-4 209 214 10.1080/13638490310001642054 14713587

[B5] Weiss HR Weiss G Schaar HJ Incidence of surgery in conservatively treated patients with scoliosis Pediatr Rehabil 2003 6 2 111 118 10.1080/13638490310001593446 14534048

[B6] Maruyama T Kitagawa T Takeshita K Mochizuki K Nakamura K Conservative treatment for adolescent idiopathic scoliosis: can it reduce the incidence of surgical treatment? Pediatr Rehabil 2003 6 3-4 215 219 10.1080/13638490310001642748 14713588

[B7] Negrini S Atanasio S Zaina F Romano M Parzini S Negrini A End-growth results of bracing and exercises for adolescent idiopathic scoliosis. Prospective worst-case analysis Stud Health Technol Inform 2008 135 395 408 18401107

[B8] Richards BS Bernstein RM D'Amato CR Thompson GH Standardization of criteria for adolescent idiopathic scoliosis brace studies: SRS Committee on Bracing and Nonoperative Management Spine 2005 30 18 2068 2075 discussion 2076-2067 10.1097/01.brs.0000178819.90239.d0 16166897

[B9] Negrini S Grivas TB Kotwicki T Rigo M Zaina F Guidelines on "Standards of management of idiopathic scoliosis with corrective braces in everyday clinics and in clinical research": SOSORT Consensus 2008 Scoliosis 2009 4 1 2 10.1186/1748-7161-4-19 19149877 PMC2651850

[B10] Janicki JA Poe-Kochert C Armstrong DG Thompson GH A comparison of the thoracolumbosacral orthoses and providence orthosis in the treatment of adolescent idiopathic scoliosis: results using the new SRS inclusion and assessment criteria for bracing studies J Pediatr Orthop 2007 27 4 369 374 17513954 10.1097/01.bpb.0000271331.71857.9a

[B11] Coillard C Vachon V Circo AB Beausejour M Rivard CH Effectiveness of the SpineCor brace based on the new standardized criteria proposed by the scoliosis research society for adolescent idiopathic scoliosis J Pediatr Orthop 2007 27 4 375 379 17513955 10.1097/01.bpb.0000271330.64234.db

[B12] Coillard C Circo AB Rivard CH Effectiveness of the SpineCor brace based on the standardized criteria proposed by the S.R.S. for adolescent idiopathic scoliosis - up to date results6th International Conference on Conservative Management of Spinal Deformites: 2009; Lyon 2009 saa15

[B13] Negrini S The Evidence-Based ISICO Approach to Spinal Deformities 2007 1 Milan, Boston: ISICO

[B14] Sibilla P Negrini S, Rainero G Trent'anni di scoliosi. Lezione "non" magistrale Rachide & Riabilitazione 2002 2002 1 Vigevano: Gruppo di Studio Scoliosi e patologie vertebrali 73 92

[B15] Sibilla P Negrini S, Sibilla P Il trattamento conservativo attivo della scoliosi idiopatica in Italia Le deformità vertebrali: stato dell'arte 2001 2 Vigevano: Gruppo di Studio Scoliosi e patologie vertebrali 20 41

[B16] Zaina F Negrini S Atanasio S Fusco C Romano M Negrini A Specific exercises performed in the period of brace weaning can avoid loss of correction in Adolescent Idiopathic Scoliosis (AIS) patients: Winner of SOSORT's 2008 Award for Best Clinical Paper Scoliosis 2009 4 1 8 10.1186/1748-7161-4-8 19351395 PMC2672077

[B17] Negrini S Atanasio S Negrini F Zaina F Marchini G The Sforzesco brace can replace cast in the correction of adolescent idiopathic scoliosis: A controlled prospective cohort study Scoliosis 2008 3 1 15 10.1186/1748-7161-3-15 18976485 PMC2612643

[B18] Negrini S Marchini G Efficacy of the symmetric, patient-oriented, rigid, three-dimensional, active (SPoRT) concept of bracing for scoliosis: a prospective study of the Sforzesco versus Lyon brace Eura Medicophys 2007 43 2 171 181 discussion 183-174 16955065

[B19] Negrini S Zaina F Romano M Negrini A Parzini S Specific exercises reduce brace prescription in adolescent idiopathic scoliosis: A prospective controlled cohort study with worst-case analysis J Rehabil Med 2008 40 6 451 455 10.2340/16501977-0195 18509560

[B20] Negrini A Negrini S Romano M Verzini N Parzini S Monticone M Negrini A A blind radiographic controlled study on the efficacy of Active Self-Correction according to SEAS.02 3rd International Conference on Conservative Management of Spinal Deformities: 7-8 April 2006 2006; Poznan (Poland): SOSORT (Society on Scoliosis Orthopaedic and Rehabilitation Treatment) 2006

[B21] Negrini S Negrini A Romano M Verzini N Parzini S A controlled prospective study on the efficacy of SEAS.02 exercises in preparation to bracing for idiopathic scoliosis Stud Health Technol Inform 2006 123 519 522 17108479

[B22] Romano M Carabalona R Petrilli S Sibilla P Negrini S Forces exerted during exercises by patients with adolescent idiopathic scoliosis wearing fiberglass braces Scoliosis 2006 1 12 10.1186/1748-7161-1-12 16859544 PMC1578587

[B23] Negrini S Grivas TB Kotwicki T Maruyama T Rigo M Weiss HR Why do we treat adolescent idiopathic scoliosis? What we want to obtain and to avoid for our patients. SOSORT 2005 Consensus paper Scoliosis 2006 1 4 10.1186/1748-7161-1-4 16759352 PMC1475888

[B24] Zaina F Atanasio S Negrini S Clinical evaluation of scoliosis during growth: description and reliability Stud Health Technol Inform 2008 135 125 138 18401086

[B25] Grosso C Negrini S Boniolo A Negrini AA The validity of clinical examination in adolescent spinal deformities Stud Health Technol Inform 2002 91 123 125 15462010

[B26] Zaina F Negrini S Atanasio S TRACE (Trunk Aesthetic Clinical Evaluation), a routine clinical tool to evaluate aesthetics in scoliosis patients: development from the Aesthetic Index (AI) and repeatability Scoliosis 2009 4 1 3 10.1186/1748-7161-4-3 19154604 PMC2654427

[B27] Landauer F Wimmer C Behensky H Estimating the final outcome of brace treatment for idiopathic thoracic scoliosis at 6-month follow-up Pediatr Rehabil 2003 6 3-4 201 207 10.1080/13638490310001636817 14713586

[B28] Weiss HR Weiss GM Brace treatment during pubertal growth spurt in girls with idiopathic scoliosis (IS): a prospective trial comparing two different concepts Pediatr Rehabil 2005 8 3 199 206 16087554 10.1080/13638490400022212

[B29] Coillard C Circo A Rivard CH A new concept for the non-invasive treatment of Adolescent Idiopathic Scoliosis: the Corrective Movement principle integrated in the SpineCor System Disabil Rehabil Assist Technol 2008 3 3 112 119 10.1080/17483100801903913 18465393

